# Dual-Task Tests Predict Conversion to Dementia—A Prospective Memory-Clinic-Based Cohort Study

**DOI:** 10.3390/ijerph17218129

**Published:** 2020-11-03

**Authors:** Hanna B Åhman, Lars Berglund, Ylva Cedervall, Lena Kilander, Vilmantas Giedraitis, Kevin J. McKee, Martin Ingelsson, Erik Rosendahl, Anna Cristina Åberg

**Affiliations:** 1 Department of Public Health and Caring Sciences, Geriatrics, Uppsala University, SE-751 22 Uppsala, Sweden; lars.berglund@pubcare.uu.se (L.B.); ylva.cedervall@pubcare.uu.se (Y.C.); lena.kilander@pubcare.uu.se (L.K.); vilmantas.giedraitis@pubcare.uu.se (V.G.); martin.ingelsson@pubcare.uu.se (M.I.); anna.cristina.aberg@pubcare.uu.se (A.C.Å.); 2 School of Education, Health and Social Studies, Dalarna University, SE-791 88 Falun, Sweden; kmc@du.se; 3 Department of Community Medicine and Rehabilitation, Physiotherapy, Umeå University, SE-901 87 Umeå, Sweden; erik.rosendahl@umu.se

**Keywords:** dual-task, dementia, mild cognitive impairment, subjective cognitive impairment, gait

## Abstract

The aim of this study was to investigate whether Timed Up-and-Go (TUG) dual-task (TUGdt) tests predict dementia incidence among patients with subjective or mild cognitive impairment (SCI; MCI). Other study objectives were to determine whether TUGdt improves dementia prediction compared to a) demographic characteristics and standard cognitive tests alone; and b) TUG and Verbal Fluency performed separately. Patients (*n* = 172, age range 39–91 years, 78 women) with SCI or MCI performed TUGdt tests, including 1) naming animals and 2) reciting months backwards, and clinical cognitive tests at baseline. Diagnoses were identified at follow-up after 2.5 years. Logistic regression was used to predict dementia incidence, receiver operating characteristic (ROC) curves and c-statistics for predictive capacity. Analyses were stratified by age and gender. At follow-up, 51 patients had developed dementia. The TUGdt result “animals/10 s” was associated with dementia incidence (standardized odds ratio (OR) = 4.06, 95% confidence interval (CI) 2.28–7.23, *p* < 0.001), more so among patients under the median age of 72 years (standardized OR = 19.4, 95% CI 3.53–106.17, *p* < 0.001). TUGdt “animals/10 s” improved dementia prediction compared to demographic characteristics and standard tests alone (c-statistics 0.88 to 0.94) and single-task tests (c-statistics 0.86 to 0.89), but only in the younger patient group. TUGdt has the potential to become a useful tool for dementia prediction.

## 1. Introduction

The prevalence of dementia disorders is estimated to escalate given the globally aging population [1]. Age is the most significant risk factor for dementia [2], although dementia may develop as early as the fourth decade of life [3]. Among people younger than 65 years, the diagnostic procedure may be more challenging than among people with a higher age at onset, due to a wider range of mild symptoms [4,5].

The pathology of neurodegenerative dementia disorders progresses slowly, and minor cognitive decline precedes the stage of manifest dementia, i.e., when the cognitive impairment results in functional impairment. The diagnoses subjective cognitive impairment (SCI) and mild cognitive impairment (MCI) may precede a dementia diagnosis [6,7]. SCI involves a perceived reduction of cognitive function, but test results are within normal limits [8], while MCI implicates measurable cognitive deficits that do not influence everyday life activities [9]. The annual conversion rate from SCI to MCI is approximately 7% [10] and that from MCI to dementia is 10% to 15% [11]. Early identification of who among individuals with SCI or MCI will develop dementia gives an opportunity to target interventions for symptom relief or treatment of modifiable risk factors [12]. 

Current methods to identify dementia include an evaluation of the patient’s medical history, blood examination, clinical cognitive tests, structural brain imaging, cerebrospinal fluid analysis, and positron emission tomography. These methods are either time-consuming, costly, and/or invasive, and the two latter methods are generally only performed when an extended memory assessment is required. In the initial stage of identifying dementia, a basic memory assessment is normally carried out. The Swedish National Board of Health and Welfare states that the Mini Mental State Examination (MMSE) [13] and the Clock Drawing test [14] should be part of a basic memory assessment [15]. Similar procedures apply also for other countries, with MMSE and the Clock Drawing test being two of the most widely used tests for diagnosing dementia [14,16]. Other cost-effective and non-invasive tools that allow identification of individuals at the preclinical or early stages of dementia have been called for [17]. Among such tests, dual-task testing (e.g., gait combined with a verbal task) has shown promise in previous studies [17,18]. This is explained by the notion that motor control and cognition partly share common brain networks [19], and dual-tasking may provoke an overload in these networks [20]. Additionally, advanced age may limit the ability to dual-task [21]. Since walking requires a higher level of attention with advancing age [22], stronger effects of walking on cognitive performance have been shown among older than among younger people during dual-task testing [23,24]. 

Recently, a limited number of longitudinal studies have presented dual-task test outcomes that show potential regarding dementia prediction [18,25]. Dual-task tests that involve straight-line walking have shown that the dual-task cost (calculated in this case as the relative difference in gait speed between single- and dual-task performance) predicts conversion to dementia among individuals with MCI [18]. Moreover, reduced dual-task gait speed and increased gait variability measured with an accelerometer have shown potential to predict progression from MCI to Alzheimer’s disease [25]. In these studies, specialized electronic equipment (an electronic walkway with embedded pressure sensors [18] or an accelerometric method including an acceleration sensor, a recording device, and a computer program [25]) was required, which may imply that the clinical usefulness of these tests is limited. The mobility test, Timed Up-and-Go (TUG single-task, TUGst), has been used without sophisticated electronic equipment in a few dual-task studies instead of straight-line walking [26,27,28,29]. The TUGst is widely used in geriatric medicine to evaluate functional mobility through observation and timing of a movement sequence, which includes walking and transitions [30]. To our knowledge, the only previous longitudinal dual-task study that involves TUGst found that dual-task cost (calculated in this case as the relative time difference between single- and dual-task performance) does not predict conversion from MCI to dementia [26]. 

Our aim was to investigate whether an easy-to-administer and inexpensive dual-task test, i.e., TUG dual-task (TUGdt) can be useful in the initial phase of memory assessments, by exploring whether TUGdt test outcomes can predict dementia incidence among patients with SCI or MCI, and whether these outcomes can improve predictive capacity compared to demographic characteristics and standard cognitive test results alone (i.e., MMSE and Clock Drawing test). Additionally, we aimed to investigate whether our TUGdt test that is based on the two original single-task tests, TUGst [30] and Verbal Fluency test (naming animals) [31], can improve predictive capacity compared to the two single-task tests performed separately.

## 2. Methods

### 2.1. Setting and Participants 

The current study forms part of the Uppsala–Dalarna Dementia and Gait (UDDGait) project [28,32,33]. UDDGait is an ongoing, longitudinal cohort project with the overall aim of investigating dual-task test outcomes as markers for dementia disorders.

Participants were included consecutively when undergoing assessment at two specialist memory clinics in Sweden. The exclusion criteria were as follows: inability to walk three meters back and forth or to rise from a sitting position, indoor use of a walking aid, current or recent hospitalization (within the last two weeks), and need of an interpreter to communicate in Swedish. In total, 172 patients from UDDGait participated in the current study, i.e., patients who were diagnosed with SCI or MCI at baseline and from whom diagnostic information was possible to attain 2.5 years after baseline. Ethical approval was granted from the Regional Ethical Review Board in Uppsala. Informed consent was attained from all participants during enrollment.

### 2.2. Data Collection

#### 2.2.1. Data Collection at Baseline

The data collection procedures used in UDDGait have been described in detail previously [28,32,33]. All participants reported demographic characteristics including educational level (university education or not) and marital status. The TUGst and TUGdt tests were performed according to a standardized procedure (see below, Section 2.2.2., Timed Up-and-Go single- and dual-task tests). For descriptive purposes, participants carried out a short version of the General Motor Function Assessment Scale [34], a balance test according to Bohannon [35], and assessment of handgrip strength using a dynamometer [36]. Additionally, participants were assessed for depressive symptoms using the 4-item Geriatric Depression Scale [37]. The MMSE [13] and the neurocognitive test 7 Minute Screen [38] were carried out as part of the memory assessment. From the 7 Minute Screen, results from the Clock Drawing test [39] and the Verbal Fluency test [31] (naming as many animals as possible in 60 s) were used in the current study. As a result of the memory assessment and blinded to the TUG performances, geriatricians made diagnoses of SCI, MCI, and dementia based on established criteria [6,40,41,42,43,44]. The diagnoses were collected from medical records after the baseline data collection had been completed. 

#### 2.2.2. Timed Up-and-Go Single- and Dual-Task Tests

The mobility test TUGst involves the test person rising from an armchair, walking three meters at a comfortable pace, turning around at a mark on the floor, walking back, and sitting down again. The test is timed from the individual’s back leaving the backrest to sitting down (their posterior touching the seat) [30]. The TUGdt test procedure used in the current study has previously been tested and developed [33]. The testing was performed in the following order: TUGst, TUGdt naming animals (TUGdt NA), and TUGdt reciting months backwards (TUGdt MB). The test order was chosen to represent increasing difficulty. The physical therapist who led the testing gave standardized instructions to the participant before each test. TUGdt NA included naming different animals while completing TUGst. TUGdt MB included reciting months in reverse order, starting with the last month of the year, while completing TUGst. The participants were instructed to complete all tests at their own speed, concerning both mobility and verbal performance, and if they did not know what to say, they were asked to complete the mobility sequence. The tests were timed with a stopwatch to an accuracy of 0.01 s, and video was recorded with two ordinary cameras. 

### 2.3. Review of Medical Records

The participants’ medical records were reviewed up to 2.5 years after baseline. Participants were classified as having “converted” to dementia after receiving such a diagnosis, and as “not converted” to dementia when a diagnosis of SCI or MCI had been confirmed at least 1.5 years after baseline, or when a reversion to normal cognition had been stated (*n* = 62), all based on established criteria [6,40,41,42,43,44]. For participants who had not been re-evaluated at one of the specialist memory clinics, a geriatrician with many years’ experience of performing memory assessments reviewed their primary care medical records and using the same established criteria found evidence for conversion or non-conversion to dementia in 65 participants. Among participants whose medical records did not provide sufficient information, MMSE scores from a follow-up visit at two years after baseline were used to rule out conversion to dementia. A score that was higher, unchanged, or a maximum of one point less compared with baseline was considered to signify non-conversion [45] (*n* = 45). The geriatrician who performed these reviews was blinded to the baseline TUG test results.

### 2.4. Data Preparation and Statistical Analyses

#### Data Preparation

The video cameras’ sound recordings were used to evaluate the verbal performance. The number of different animals during TUGdt NA, and the number of months in correct order during TUGdt MB were counted. The number of words were then validated by another researcher. When there were uncertainties, the sound recordings were reassessed until consensus between assessors was obtained. Each participant’s average number of words recited per 10 s during the TUGdt tests was calculated as 10 × (TUGdt number of words/TUGdt time score). Dual-task cost was calculated as 100 × (TUGdt time score−TUGst time score)/TUGst time score. 

### 2.5. Statistical Analyses

Analyses were carried out using SPSS version 25 (IBM Corp., Armonk, NY, USA) and SAS® version 9.4 (SAS Institute Inc., Cary, NC, USA). Participants’ baseline characteristics were summarized using means and standard deviations or frequencies and percentages. The test results were not normally distributed and are therefore presented as medians with interquartile ranges. 

Logistic regression models were used to assess the ability of TUG test outcomes to predict dementia incidence. Univariate logistic regression models were used to calculate odds ratios (OR) for the separate TUG test outcomes and dementia incidence at follow-up (Model 1). The covariates age (continuous variable), gender, and educational level were added (Model 2). In Model 3, the MMSE score and Clock Drawing score (dichotomized: 1–6 points or 7 points, signifying inadequate or adequate performance) were added to Model 2. Results were expressed as standardized ORs (sORs) with 95% confidence intervals. For all time scores including TUGdt costs, the sORs express the increase of odds per one standard deviation increase of the variable. For the number of animals and months, as well as “animals/10 s” and “months/10 s” the sORs express the increase of odds per one standard deviation decrease of the variable. Tests of effect modification by age (continuous variable) and gender on associations between TUG variables and dementia incidence were performed in the adjusted models. All analyses were carried out in the total sample and, when the corresponding effect modification test was statistically significant, stratified by age (under the median age/median age and above) and by gender (female/male). Statistical tests were two-tailed and the significance level was set at *p* < 0.05. 

Receiver operating characteristic (ROC) curves were constructed, and areas under the curves (AUC, c-statistics) were used to determine predictive capacity. According to Hosmer et al [46] c-statistics of 0.5–0.7 are poor, 0.7–0.8 acceptable, 0.8–0.9 excellent, and 0.9–1.0 outstanding. Incremental ROC curves were completed to show the predictive capacity of the TUGdt test outcomes when added to a model including age, gender, educational level, MMSE score, and Clock Drawing test score (Model 3). Incremental ROC curves were also completed in order to show whether TUGdt NA improves predictive capacity compared to the two original single-task tests performed separately (i.e., TUGst and Verbal Fluency).

Since the investigated sample comprised two diagnostic groups at baseline (SCI and MCI), a sensitivity analysis was carried out in which individuals with SCI were excluded. This analysis did not materially change any of the results.

## 3. Results

### 3.1. Participant Characteristics and Conversion to Dementia

The 172 participants were aged between 39 and 91 years at baseline (mean 71 years; 45.3% female). Of these, 61 individuals were diagnosed with SCI and 111 with MCI. At follow-up, a total of 51 participants (30%) had converted to dementia, of which 49 had MCI and two had SCI at baseline. Among the participants who converted to dementia, 26 developed AD, 10 unspecified dementia, four Parkinson’s dementia, three vascular dementia, three dementia with Lewy bodies, three frontotemporal dementia, and two AD/vascular dementia. 

Baseline characteristics and cognitive test results in the total sample, as well as stratified according to conversion to dementia, are summarized in Table 1. All participants completed TUGdt NA, whereas two participants discontinued TUGdt MB. Motor function test results are presented in Supplementary Table S1. The study sample was stratified by age (under the median age/median age and above). Among participants younger than 72 years (*n* = 84), 12 individuals converted to dementia (14%), and among participants aged 72 years or older (*n* = 88), 39 individuals converted to dementia (44%). Table 2 presents baseline characteristics and test results stratified by age and by conversion to dementia.

### 3.2. Prediction of Dementia Incidence

Univariate logistic regression analyses (Model 1) showed significant associations between all included baseline TUG test outcomes and dementia incidence, except for the dual-task cost measures (Table 3). After adjusting for age, gender, and educational level (Model 2), most associations remained significant: TUGst time score (OR = 1.93, 95% CI 1.16–3.20, *p* = 0.011), TUGdt NA time score (OR = 1.93, 95% CI 1.15–3.21, *p* = 0.012), TUGdt NA number of animals (OR = 1.96, 95% CI 1.24–3.10, *p* = 0.004), TUGdt NA animals/10 s (OR = 3.14, 95% CI 1.70–5.81, *p* < 0.001), TUGdt MB number of months (OR = 1.56, 95% CI 1.09–2.32, *p* = 0.029), and TUGdt MB months/10 s (OR = 2.05, 95% CI 1.23–3.39, *p* = 0.006). 

**Figure 1:**
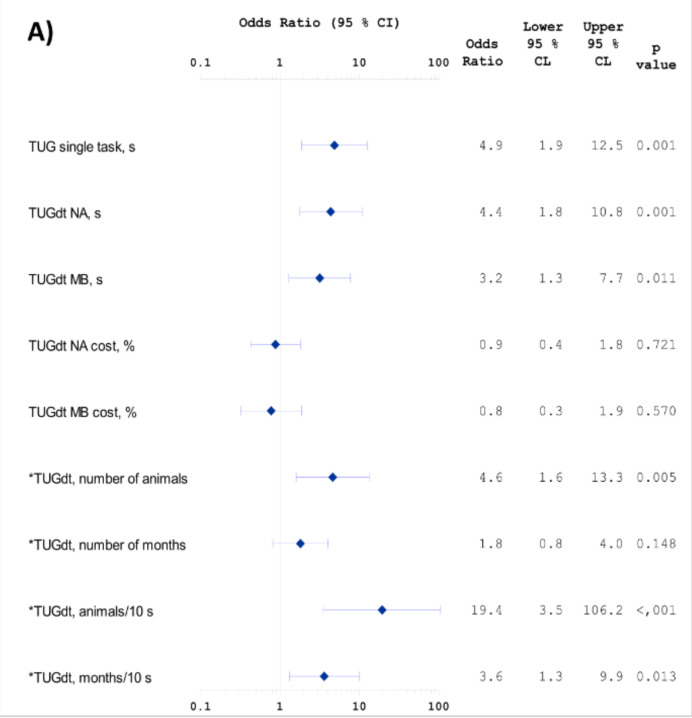

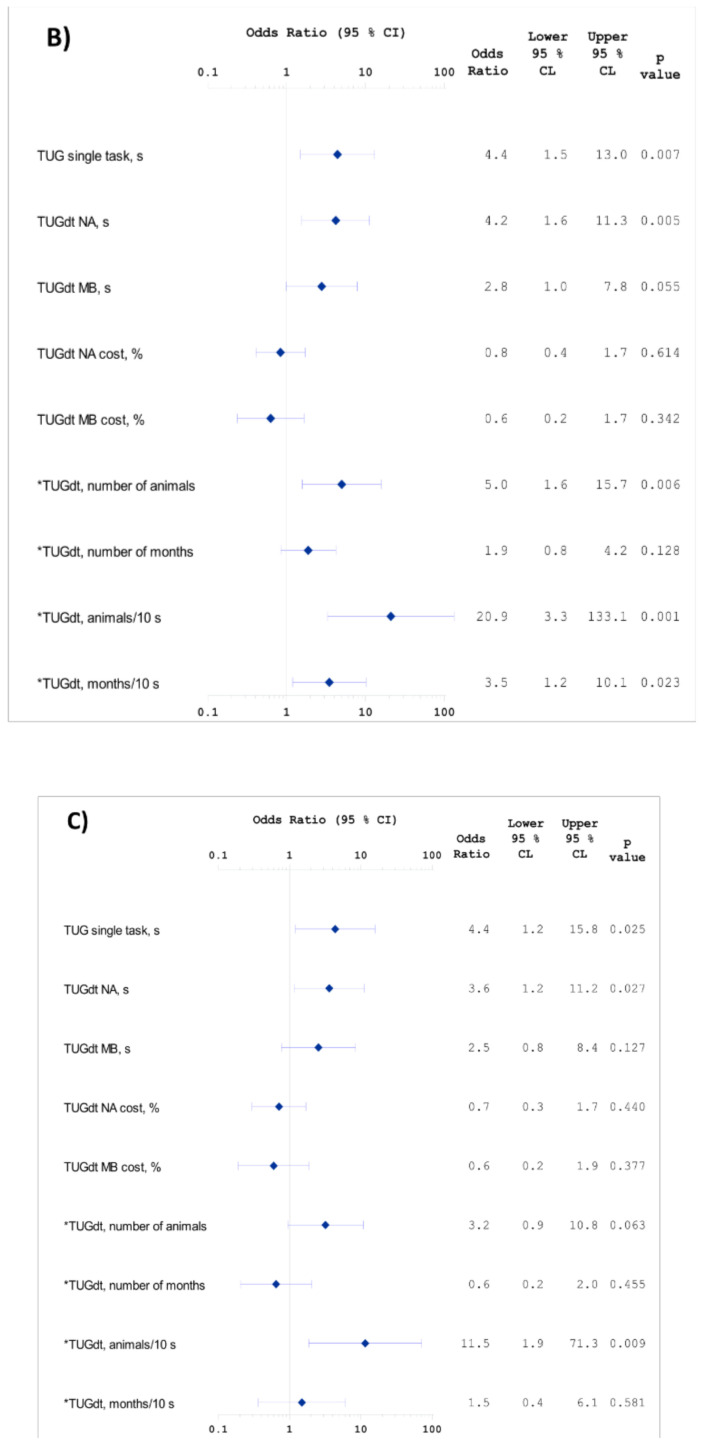
Standardized odds ratios for conversion to dementia among participants younger than 72 years. Forest plot presenting standardized odds ratios (**A**) unadjusted (Model 1), (**B**) adjusted for age, gender, and educational level (Model 2), and (**C**) adjusted for age, gender, educational level, Mini Mental State Examination, and Clock Drawing score (Model 3). Standardized odds ratios measure the increase of odds per one standard deviation increase of the predictor. * Standardized odds ratios measure the increase of odds per one standard deviation decrease of the predictor.

There was a strong effect modification on “animals/10 s” by age as a continuous variable in Models 2 and 3 (*p* = 0.004 and 0.007, respectively). Among participants younger than 72 years, the association between “animals/10 s” and dementia incidence was in Model 1: OR = 19.4, 95% CI 3.53–106.17, *p* < 0.001, in Model 2: OR = 20.9, 95% CI 3.29–133.13, *p* = 0.001, and in Model 3: OR = 11.5, 95% CI 1.9–71.3, *p* = 0.009 (Figure 1). Among the younger participants, all models presented several significant associations in addition to “animals/10 s” (Figure 1). No effect modification was found on “animals/10 s” by gender (*p* = 0.37).

Among the investigated dual-task test outcomes, “animals/10 s” presented the highest predictive capacity in the total sample, unadjusted c-statistics = 0.76. The c-statistics were 0.89 for “animals/10 s” among younger participants and 0.64 among older participants. With adjustment for age, gender, and educational level, the c-statistics were 0.80 in the total sample, 0.90 among younger participants, and 0.66 among older participants.

### 3.3. Improvement of Predictive Capacity Based on Demographic Characteristics and Standard Cognitive Tests

**Figure 2:**
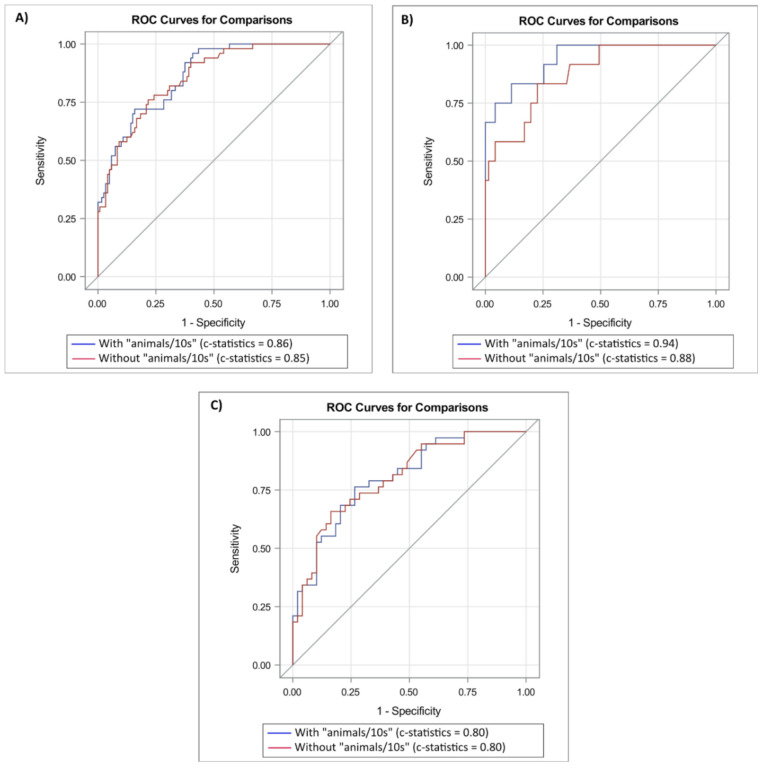
Prediction of dementia incidence. Receiver operating characteristic (ROC) curves presenting the predicting capacity (c-statistics) of “animals/10 s” (blue curve) added to a base model of age, gender, educational level, Mini Mental State Examination score, and Clock Drawing score (red curve) in (**A**) the total sample, (**B**) participants aged less than 72 years, and (**C**) participants aged 72 years and older.

With adjustment for age, gender, educational level, MMSE, and Clock Drawing score (Model 3), there were no significant associations in the total sample between any TUG test outcomes and dementia incidence (Table 3). However, among participants younger than 72 years, the following TUG test outcomes showed significant associations: TUGst time score (OR = 4.36, 95% CI 1.20–15.77, *p* = 0.025), TUGdt NA time score (OR = 3.60, 95% CI 1.16–11.21, *p* = 0.027), and TUGdt NA “animals/10 s” (OR = 11.50, 95% CI 1.86–71.25, *p* = 0.009). Among participants aged 72 years or older, Model 3 did not result in any significant associations. Incremental ROC-curve analyses showed that in the total sample, “animals/10 s” added marginal value to Model 3 (0.85 to 0.86, *p* = 0.067) (Figure 2). Among younger participants, “animals/10 s” increased the c-statistic from 0.88 to 0.94 (*p* = 0.009). Among older participants, “animals/10 s” did not add any value to the same model (0.80 to 0.80, *p* = 0.845).

### 3.4. Added Predictive Capacity of a Dual-Task Test to Two Single-Task Tests

When investigating whether TUGdt NA improves the predictive capacity compared to the combination of the two original single-task tests it is based on, i.e., TUGst time score and Verbal Fluency score, adding “animals/10 s” did not change the c-statistic significantly in the total sample. However, among patients younger than 72 years, the c-statistics increased from 0.86 to 0.89 (*p* = 0.025). The corresponding changes in c-statistics were not significant among the older patients.

## 4. Discussion

We present novel findings concerning the use of dual-task testing for prediction of conversion to dementia. A strong association between the TUGdt outcome “animals/10 s” and dementia incidence adjusted for demographic characteristics was found in the total sample (OR = 3.1) and particularly among participants younger than 72 years (OR = 20.9). “Animals/10 s” presented the highest predictive capacity in the total sample with an acceptable unadjusted c-statistic of 0.76, while an excellent predictive capacity for “animals/10 s” was found among younger participants, where the c-statistic was 0.89. Moreover, the TUGdt NA improved a model including demographic characteristics and standard cognitive test results among the younger participants by increasing the c-statistic from 0.88 to 0.94. Additionally, we showed that TUGdt NA has a greater capacity for predicting dementia conversion among the younger participants compared to the two original single-task tests that TUGdt NA is based on, i.e., TUGst time score and Verbal Fluency score. 

The investigated dual-task test outcomes were less predictive of dementia incidence among the older participants than among the younger participants in our sample. The ability to dual-task is altered by age; walking is a less automatic task for older than for younger adults [22,47], and the effect of walking on cognitive performance is much stronger in older people [23]. Additionally, an age-related decrease of brain mass, particularly in the frontal lobe, contributes to a decline of cognitive processing capacity, which may affect the ability to dual-task [21]. In summary, advanced age may entail other central alterations that make the TUGdt test less sensitive for predicting conversion to dementia. The strong associations found regarding younger participants are interesting in terms of the clinical usefulness of TUGdt. The time required for a diagnosis of young-onset dementia (<65 years) has been found to be greater than that for late-onset dementia [4], probably due to the wide range of symptoms, together with low expectations that dementia might be the underlying cause. The current sample was additionally stratified by gender, however no gender differences regarding the associations between TUGdt test outcomes and dementia incidence were found.

The TUGdt outcome “animals/10 s”, where the performances of the verbal and mobility tasks are combined in one measure, was the most accurate outcome in predicting dementia incidence in the present study. To our knowledge, “words per time unit” has not been used for this purpose before in longitudinal dual-task studies. However, in our previous cross-sectional study, as well as in studies by other researchers, “words per time unit” has been found to differentiate between dementia, MCI, SCI, and healthy controls [32,48,49]. Moreover, we have previously shown that “animals/10 s” correlates with neurodegeneration based on cerebrospinal fluid biomarkers [28]. 

Dual-task cost did not predict conversion to dementia in the current study. In contrast, in a previous study in which straight-line walking was used, dual-task cost showed potential for predicting dementia incidence [18]. A possible reason for these divergent results is that TUGst places higher demands on executive function than straight-line walking does, which may entail smaller differences between single- and dual-task time scores. Another possible explanation may be related to the priority of the mobility task in the current study, which most likely reduced the time scores. In line with our results, a previous dual-task study based on TUGst did not find dual-task cost predictive of conversion to dementia [26]. In that study, the participants were instructed to walk “as fast and safe as possible”, which probably influenced the participants to prioritize walking above the verbal task.

Since TUGdt NA is a test based on two tasks, and data for both were collected as original single-task tests in the current study (TUGst and Verbal Fluency test), we aimed to investigate whether our TUGdt test improves dementia prediction compared to the two single-task tests. The adding of “animals/10 s” to TUGst time score and Verbal Fluency score increased the c-statistic among patients younger than 72 years, which supports the theory that two simultaneously performed tasks interfere with each other and compete for cortical resources [50]. The value of performing a dual-task test instead of one of its single-task components has been shown previously [18]. However, no incremental analyses including the separate original single-task tests have been presented before.

Our study was carried out in a clinical environment, using the standard cognitive tests that are part of the basic memory assessment set by The Swedish National Board of Health and Welfare. For that reason, other tests that might be more accurate in memory assessment, such as the Montreal Cognitive Assessment (MoCA) [51], were not used. The diagnoses of SCI and MCI are common at memory clinics and both were therefore included in our study. In the event, only two of our participants with a baseline diagnosis of SCI progressed to dementia, probably due to the relatively short follow-up time of 2.5 years. However, the follow-up time was considered generous enough to allow for disease progression in a sufficient number of individuals to explore the differences in dual-task test performance between converters and non-converters. Moreover, excluding patients with SCI from the analyses did not change the associations between TUG test outcomes and dementia incidence.

The current study has limitations that should be taken into consideration. There were relatively few participants who converted to dementia in the younger age group resulting in wide confidence intervals. Additionally, the division of age groups of younger (39–71 years) and older (72–91 years) participants was by median split. An optimal cutoff age was not explored due to the limited sample size. However, ongoing UDDGait studies will be focused on the predictive value of TUGdt NA after four and eight years, when more individuals can be expected to have converted to dementia. Another limitation concerns the reliability of diagnoses at follow-up, given that not all participants had been re-evaluated at the memory clinics. However, medical records were carefully reviewed for evidence of conversion or non-conversion to dementia by a geriatrician with many years’ experience of memory assessments, using established diagnostic criteria. An additional issue is that the educational level in the sample is likely to be higher than that in the target population due to the data being collected in university cities, which restricts the extent to which the study findings can be generalized. Furthermore, educational level and cognitive reserve could influence the symptom progress [52], but the variable used in the current study (university education or not) may not be nuanced enough to fully capture potential confounding. The order in which the two dual-task tests were performed was believed to represent increasing task difficulty, but optionally the tasks could have been randomized to avoid the risk of learning or fatigue effects. 

The study also has several strengths, including the standardized TUG test procedures and the subsequent validation of the verbal performances via video recordings. Additionally, the tests were carried out in a clinical environment without the requirement of specialized or expensive equipment, which suggests a potential for implementing the tests in such settings. 

## 5. Conclusions

Among patients younger than 72 years with SCI or MCI, the TUGdt outcome “animals/10 s” predicted dementia incidence and improved models with demographic characteristics and standard cognitive tests regarding the prediction of conversion to dementia. These findings were not apparent in patients aged 72 years and above. Our results indicate that TUGdt NA has the potential to be used as an easy-to-administer and inexpensive tool for dementia prediction among younger patients in the initial phase of a memory assessment. 

## Figures and Tables

**Table 1:** Participant characteristics and test results at baseline in the total sample and stratified according to conversion to dementia.

Characteristic	Total Sample; SCI or MCI (*n* = 172)	Converted to Dementia (*n* = 51)	Did not Convert to Dementia (*n* = 121)
Age, mean ± SD (min-max)	71.0 ± 8.7 (39–91)	75.7 ± 7.3 (56–91)	69.0 ± 8.6 (39–88)
Age groups, *n* (%)			
30–39 years	1 (0.6)	0 (0)	1 (0.8)
40–49 years	1 (0.6)	0 (0)	1 (0.8)
50–59 years	14 (8.1)	2 (3.9)	12 (9.9)
60–69 years	47 (27.3)	6 (11.8)	41 (33.9)
70–79 years	84 (48.8)	27 (52.9)	57 (47.1)
≥80 years	24 (15.7)	16 (31.4)	9 (7.4)
Female, *n* (%)	78 (45.3)	28 (54.9)	50 (41.3)
University education, *n* (%)	72 (41.9)	21 (41.2)	51 (42.1)
Married or cohabiting, *n* (%)	114 (66.3)	32 (62.7)	82 (67.8)
Test result			
MMSE	27 (25–29)	25 (23–27)	28 (26–29)
Clock Drawing	7 (6–7)	6 (4–7)	7 (6–7)
Verbal Fluency	17 (13–23)	14 (12–18)	19 (14–24)
Depressive symptoms *, *n* (%)	40 (23.3)	11 (21.6)	29 (24.0)
TUG single-task, s	12.0 (10.2–14.2)	13.5 (11.5–16.2)	11.3 (9.9–13.1)
TUGdt NA, s	13.3 (11.3–15.9)	15.2 (13.3–18.2)	12.3 (11.0–14.9)
TUGdt NA cost, %	11.4 (2.3–18.2)	9.9 (2.2–24.7)	12.3 (2.5–17.7)
TUGdt NA, number of animals	6.0 (5.0–7.3)	5.0 (4.0–7.0)	6.0 (5.0–8.0)
TUGdt NA, animals/10 s	4.5 (3.3–6.1)	3.3 (2.4–4.4)	5.2 (3.7–6.4)
TUGdt MB, s	13.5 (11.5–16.5)	14.8 (13.0–19.1)	12.8 (10.9–15.6)
TUGdt MB cost, %	13.3 (3.1–27.7)	18.6 (2.2–26.2)	12.5 (3.3–28.7)
TUGdt MB, number of months	7.0 (4.0–9.0)	5.5 (3.0–8.3)	7.0 (5.9–9.0)
TUGdt MB, months/10 s	4.8 (2.9–6.8)	3.3 (1.8–5.4)	5.2 (3.6–7.6)

Baseline characteristics and test results are presented as medians and interquartile range if not stated otherwise. SCI = subjective cognitive impairment; MCI = mild cognitive impairment; SD = standard deviation; MMSE = Mini Mental State Examination; TUG = Timed Up-and-Go; TUGdt = Timed Up-and-Go dual-task; NA = naming animals; MB = months backwards. * Depressive symptoms defined as two points or more according to the 4-item Geriatric Depression Scale.

**Table 2:** Participant characteristics and test results at baseline stratified by age and by conversion to dementia.

Characteristic		Patients < 72 years	Patients ≥ 72 years
Number of patients, *n* (%)	Baseline SCI or MCI	84 (100)	88 (100)
	Conversion	12 (14.3)	39 (44.3)
	Non-conversion	72 (85.7)	49 (55.7)
Age, mean ± SD (min-max)	Baseline SCI or MCI	64.1 ± 6.5 (39–71)	77.6 ± 4.6 (72–91)
	Conversion	66.3 ± 5.0 (56–71)	78.6 ± 5.2 (72–91)
	Non-conversion	63.7 ± 6.6 (39–71)	76.7 ± 3.9 (72–88)
Female, *n* (%)	Baseline SCI or MCI	39 (46.4)	39 (44.3)
	Conversion	9 (75.0)	19 (48.7)
	Non-conversion	30 (41.7)	20 (40.8)
University education, *n* (%)	Baseline SCI or MCI	40 (47.6)	32 (36.4)
	Conversion	7 (58.3)	14 (35.9)
	Non-conversion	33 (45.8)	18 (36.7)
Married or cohabiting, *n* (%)	Baseline SCI or MCI	58 (69.0)	56 (63.6)
	Conversion	8 (66.7)	42 (61.5)
	Non-conversion	50 (69.4)	32 (65.3)
Test result			
MMSE, score	Baseline SCI or MCI	28 (25–29)	26 (24–28)
(Score range 0–30)	Conversion	25 (23–27)	25 (23–27)
	Non-conversion	28 (26–29)	28 (26–29)
Clock Drawing test, score	Baseline SCI or MCI	7 (6–7)	7 (6–7)
(Score range 0–7)	Conversion	6 (4–7)	6 (5–7)
	Non-conversion	7 (7–7)	7 (6–7)
Verbal Fluency test *, score	Baseline SCI or MCI	19 (13–24)	16 (12–22)
	Conversion	13 (11–18)	15 (12–17)
	Non-conversion	20 (14–25)	19 (15–23)
Depressive symptoms **, *n* (%)	Baseline SCI or MCI	26 (31.0)	14 (15.9)
	Conversion	3 (25.0)	8 (20.5)
	Non-conversion	23 (31.9)	6 (12.2)
TUG single-task, s	Baseline SCI or MCI	11.0 (9.6–12.9)	12.6 (11.1–14.5)
	Conversion	13.0 (11.9–17.0)	13.9 (11.4–16.1)
	Non-conversion	10.6 (9.6–12.6)	12.3 (10.8–13.9)
TUGdt NA, s	Baseline SCI or MCI	11.8 (10.3–15.0)	14.0 (12.3–16.6)
	Conversion	15.8 (13.0–19.0)	14.9 (13.4–17.7)
	Non-conversion	11.5 (10.2–14.2)	13.8 (12.1–15.6)
TUGdt NA cost, %	Baseline SCI or MCI	9.8 (1.3–14.9)	13.2 (4.0–23.3)
	Conversion	5.9 (1.5–25.5)	10.9 (2.3–23.6)
	Non-conversion	10.1 (1.3–14.6)	14.0 (5.3–23.2)
TUGdt NA, number of animals	Baseline SCI or MCI	6.0 (5.0–8.0)	6.0 (5.0–7.0)
	Conversion	4.5 (4.0–6.8)	6.0 (4.0–7.0)
	Non-conversion	6.5 (6.0–8.0)	6.0 (5.0–8.0)
TUGdt NA, animals/10 s	Baseline SCI or MCI	5.5 (3.6–6.7)	4.0 (2.8–5.2)
	Conversion	3.0 (2.2–3.6)	3.5 (2.4–4.4)
	Non-conversion	5.9 (4.4–6.9)	4.5 (3.4–5.6)
TUGdt MB, s	Baseline SCI or MCI	12.6 (10.8–15.4)	14.8 (12.5–17.8)
	Conversion	15.5 (12.6–18.8)	14.8 (13.4–20.4)
	Non-conversion	12.3 (10.4–14.4)	13.7 (12.4–16.6)
TUGdt MB cost, %	Baseline SCI or MCI	11.1 (3.2–21.3)	21.4 (2.3–30.7)
	Conversion	16.5 (1.2–21.6)	20.4 (2.1–31.2)
	Non-conversion	11.0 (3.3–21.3)	21.8 (2.3–30.7)
TUGdt MB, number of months	Baseline SCI or MCI	7.0 (5.0–8.0)	7.0 (4.0–9.0)
	Conversion	5.0 (3.0–7.8)	5.5 (3.0–9.0)
	Non-conversion	7.0 (5.0–8.8)	7.5 (4.3–9.0)
TUGdt MB, months/10 s	Baseline SCI or MCI	5.4 (3.7–7.3)	4.3 (2.4–6.1)
	Conversion	3.1 (2.0–5.4)	3.5 (1.7–5.4)
	Non-conversion	5.5 (4.1–7.6)	4.7 (2.8–7.6)

Baseline characteristics and test results are presented as medians and interquartile range if not stated otherwise. SCI = subjective cognitive impairment; MCI = mild cognitive impairment; SD = standard deviation; MMSE = Mini Mental State Examination; TUG = Timed Up-and-Go; TUGdt = Timed Up-and-Go dual-task; NA = naming animals; MB = months backwards. * Naming different animals during 60 s, in a sitting position. ** Depressive symptoms defined as two points or more according to the 4-item Geriatric Depression Scale.

**Table 3:** Standardized odds ratios for conversion to dementia 2.5 years after baseline in the total sample.

	Model 1		Model 2		Model 3	
	sOR (95% CI)	*p*-Value	sOR (95% CI)	*p*-Value	sOR (95% CI)	*p*-Value
TUGst time score, s	2.73 (1.74–4.30)	<0.001	1.93 (1.16–3.20)	0.011	1.49 (0.82–2.70)	0.189
TUGdt NA time score, s	2.64 (1.68–4.15)	<0.001	1.93 (1.15–3.21)	0.012	1.49 (0.83–2.67)	0.183
* TUGdt NA, number of animals	2.03 (1.31–3.15)	<0.001	1.96 (1.24–3.10)	0.004	1.41 (0.84–2.35)	0.190
* TUGdt NA, animals/10 s	4.06 (2.28–7.23)	<0.001	3.14 (1.70–5.81)	<0.001	1.89 (0.95–3.73)	0.067
TUGdt NA cost, %	1.03 (0.76–1.40)	0.840	0.99 (0.73–1.33)	0.939	0.97 (0.70–1.34)	0.866
TUGdt MB time score, s	2.16 (1.40–3.33)	<0.001	1.40 (0.85–2.31)	0.185	0.95 (0.52–1.73)	0.857
* TUGdt MB, number of months	1.56 (1.07–2.28)	0.021	1.56 (1.05–2.32)	0.029	0.86 (0.52–1.41)	0.553
* TUGdt MB, months/10 s	2.53 (1.57–4.08)	<0.001	2.05 (1.23–3.39)	0.006	0.96 (0.52–1.78)	0.893
TUGdt MB cost, %	0.89 (0.65–1.23)	0.484	0.76 (0.53–1.10)	0.142	0.62 (0.36–1.05)	0.073

Standardized odds ratios measure the increase of odds per one standard deviation increase of the predictor. * Standardized odds ratios measure the increase of odds per one standard deviation decrease of the predictor. Model 1: unadjusted. Model 2: adjusted for age, gender, and educational level. Model 3: adjusted for age, gender, educational level, Mini Mental State Examination, and Clock Drawing test. Statistically significant if *p* < 0.05, in bold. TUG = Timed Up-and-Go; sOR = standardized odds ratio; CI = confidence interval; TUGst = Timed Up-and-Go single-task; TUGdt = Timed Up-and-Go dual-task; TUGdt NA = Timed Up-and-Go dual-task naming animals; TUGdt MB = Timed Up-and-Go dual-task months backwards.

## Supplementary Materials

The following are available online at https://www.mdpi.com/1660-4601/17/21/8129/s1, Table S1: Motor function test results at baseline stratified according to conversion to dementia.
