# Supplementary material for: Dual-Task Tests Predict Conversion to Dementia—A Prospective Memory-Clinic-Based Cohort Study
Source: Int J Environ Res Public Health. 2020 Nov 3;17(21):8129. doi: 10.3390/ijerph17218129 (PMC7662628; doi:10.3390/ijerph17218129)
Supplement: Supplementary file 1 [file ijerph-17-08129-s001.pdf]

International Journal of
Environmental Research and Public Health

MDPI

Supplementary Materials: Dual-Task Tests Predict Conversion to Dementia—A Prospective Memory-Clinic-Based Cohort Study

Table S1. Motor function test results at baseline stratified according to conversion to dementia.

**Motor function test result**	**Total sample; SCI or MCI (n = 172)**	**Converted to dementia (n = 51)**	**Did not convert to dementia (n = 121)**
GMF Dependence	0 (0-0)	0 (0-0)	0 (0-0)
GMF Pain	0 (0-1)	0 (0-0)	0 (0-1)
GMF Insecurity	0 (0-0)	0 (0-0)	0 (0-0)
Balance*	5 (5-6)	5 (5-5)	6 (5-6)
Hand grip, pounds	70 (53-97)	60 (45-82)	75 (55-102)

Baseline characteristics and cognitive test results presented as medians and interquartile range if not stated otherwise. SCI = Subjective cognitive impairment; MCI = Mild cognitive impairment; GMF = General Motor Function Assessment Scale (score range 0-7). *Balance according to Bohannon (score range 0-6)
